# Real-Life Comparison of Four JAK Inhibitors in Rheumatoid Arthritis (ELECTRA-*i* Study)

**DOI:** 10.3390/jcm13061821

**Published:** 2024-03-21

**Authors:** Maurizio Benucci, Francesca Li Gobbi, Arianna Damiani, Edda Russo, Serena Guiducci, Mariangela Manfredi, Barbara Lari, Valentina Grossi, Maria Infantino

**Affiliations:** 1 Rheumatology Unit, S. Giovanni di Dio Hospital, Azienda USL-Toscana Centro, 50143 Florence, Italy; francesca.ligobbi@uslcentro.toscana.it; 2 Department of Experimental and Clinical Medicine, University of Florence, 50143 Florence, Italy; ariannadam@outlook.it (A.D.); edda.russo@unifi.it (E.R.); serena.guiducci@unifi.it (S.G.); 3 Immunology and Allergology Laboratory Unit, S. Giovanni di Dio Hospital, Azienda USL-Toscana Centro, 50142 Florence, Italy; mariangela.manfredi@uslcentro.toscana.it (M.M.); barbara.lari@uslcentro.toscana.it (B.L.); valentina2.grossi@uslcentro.toscana.it (V.G.); maria2.infantino@uslcentro.toscana.it (M.I.)

**Keywords:** rheumatoid arthritis, JAK inhibitors, real life

## Abstract

**Background:** Real-world evidence of the efficacy and adverse events of JAK inhibitor treatment (Tofacitinib, Baricitinib, Upadacitinib, and Filgotinib) in rheumatoid arthritis is still limited. **Methods:** We studied 115 patients from the Rheumatology Unit of S. Giovanni di Dio Hospital affected by D2T-RA, according to the 2010 EULAR criteria. Out of the 115 patients, 17 had been treated with Baricitinib 8 mg/daily, 32 with Filgotinib 200 mg/daily, 21 with Tofacitinib 10 mg/daily, and 45 with Upadacitinib 15 mg/daily. We evaluated the clinical response after 3, 6, and 12 months of treatment and the follow-up from September 2022 to September 2023. All patients were evaluated according to the number of tender joints (NTJs), number of swollen joints (NSJs), visual analog scale (VAS), global assessment (GA), health assessment questionnaire (HAQ), Disease Activity Score (DAS28), and CDAI. Furthermore, laboratory parameters of efficacy and tolerability were evaluated. **Results:** All treatments demonstrated a statistically significant decrease in the DAS28 and CDAI scores, tender and swollen joint counts, VAS, HAQ, and patient global assessment (PGA) after 3, 6, and 12 months of treatment. All treatments showed similar behavior, and statistically significant decreases in circulating calprotectin, TNFα, and IL-6 were observed for all drugs after 12 months of treatment. In addition, soluble urokinase plasminogen activator receptor (suPAR) values showed significant differences at baseline and after 12 months of treatment for Filgotinib: 4.87 ± 4.53 vs. 3.61 ± 0.9 (0.009) and Upadacitinib: 6.64 ± 7.12 vs. 4.06 ± 3.61 (0.0003), while no statistically significant differences were found for Baricitinib: 3.4 ± 0.1 vs. 3.78 ± 0.1 and Tofacitinib: 3.95 ± 1.77 vs. 2.58 ± 0.1. The TC/HDL-C ratio (atherogenic index) showed significant differences when comparing Baricitinib vs. Filgotinib (0.0012), Filgotinib vs. Tofacitinib (0.0095), and Filgotinib vs. Upadacitinib (0.0001); furthermore, the LDL-C/HDL-C ratio in the Filgotinib group did not change (2.37 ± 0.45 vs. 2.35 ± 2.13 (NS)) after 12 months of treatment. Venous Thrombotic Events (VTEs) and major adverse cardiovascular events (MACEs) accounted for 1% of adverse events after treatment with Baricitinib. *Herpes zoster* reactivation accounted for 1% of adverse events after treatment with Filgotinib and Tofacitinib, while non-melanoma skin cancer (NMSC) accounted for 1% of adverse events after Upadacitinib treatment. **Conclusions:** Our real-world data from patients with RA show differences in some laboratory parameters and in the impact of lipid metabolism in JAK inhibitor treatment.

## 1. Introduction

Rheumatoid arthritis (RA) is a chronic autoimmune disorder characterized by local synovial and systemic inflammation, regulated by interactions among immune cells and soluble mediators (cytokines) [1]. Clinically, it presents with the involvement of both joint and non-joint tissues [1]. The current therapeutic objective is to delineate the clinical patho-phenotype by targeting the cytokine pathway, aiming to arrest the inflammatory process, prevent joint damage progression, and enhance the quality of life for the patient. In clinical practice, disease activity indices such as DAS28, CDAI, and SDAI are utilized to objectively assess patients and track specific clinical advancements [2].

The Janus kinase (JAK) signal transducers and activators of transcription (STAT) pathway constitute a critical pathogenic mechanism in the activation of the cytokine system in RA [3]. JAK inhibitors (JAKis) are small-molecule drugs that interfere with the activation of JAKs. Indeed, JAK signaling plays an essential role in the generation, differentiation, and response of immune cells through the cytokine system [4]. Through the inhibition of these signaling mechanisms, JAKs modulate immune activation, which is crucial for the development of RA across various cellular components [5]. Therefore, JAKis have emerged as an important new class of oral therapy in RA. In detail, Baricitinib (4 or 2 mg daily), Tofacitinib (5 mg twice daily), Upadacitinib (15 mg daily), and Filgotinib (200 or 100 mg daily) are currently approved for the treatment of RA by the US Food and Drug Administration (except Filgotinib) and the European Medicines Agency [6]. In 2019, JAKis were recommended as a second-line treatment for RA, at a similar level to bDMARDs (biological disease-modifying antirheumatic drugs) in terms of efficacy and safety. However, EULAR published new recommendations for the management of RA in 2023. Considering the warnings regarding cardiovascular and malignancy risks, as indicated by the findings of the ORAL Surveillance study [7], clinicians should assess cardiovascular risk factors (such as age over 65, current or past smoking history, and other cardiovascular risk factors), thromboembolic events, and neoplasms before considering the prescription of JAKis [8]. Additionally, long-term extension studies and registries have not confirmed the results of the ORAL Surveillance study [9,10]. Multiple analyses have confirmed the efficacy of JAKis, demonstrating a comparable safety profile among them [11]. Moreover, JAKis have shown efficacy in placebo-controlled studies, both when used as monotherapy and in combination with conventional synthetic DMARDs (csDMARDs), particularly with methotrexate (MTX) [12,13,14]. However, in light of recent safety concerns, there is a need for real-world data to further evaluate the safety profile of JAKis. Real-world evidence regarding the efficacy of Tofacitinib is predominantly available in countries where it has been previously introduced. Although generally reports show similar efficacy between Tofacitinib and bDMARDs [15,16], two large studies have suggested better drug persistence for Tofacitinib compared to TNFi, at least after the failure of a first biologic DMARD (bDMARD) [17,18]. Real-world evidence for Baricitinib remains limited, primarily consisting of comparisons with Tofacitinib in small-scale studies with inadequate capacity to control for confounding factors [19,20].

## 2. Materials and Methods

We studied 115 patients from the Rheumatology Unit of S. Giovanni di Dio Hospital affected by D2T-RA [21], according to the 2010 EULAR criteria [22]. Of the 115 patients, 17 had been treated with Baricitinib 8 mg/daily, 32 with Filgotinib 200 mg/daily, 21 with Tofacitinib 10 mg/daily, and 45 with Upadacitinib 15 mg/daily.

The characteristics of the study population are described in Table 1.

We evaluated the clinical response after 3, 6, and 12 months of treatment and follow-up from September 2022 to September 2023. All patients were evaluated according to the number of tender joints (NTJs), number of swollen joints (NSJs), visual analog scale (VAS), global assessment (GA), health assessment questionnaire (HAQ), Disease Activity Score (DAS28) [23], and CDAI [24] at 0, 3, 6, and 12 months. Furthermore, the following parameters were evaluated at baseline and after 3, 6, and 12 months: ESR (Alifax, Padova, Italy) and CRP levels (Unicel Coulter DxC 800 Synchron Central System; Beckman Coulter Inc, Brea, CA, USA); Anti-Citrullinated Peptide Antibodies (ACPAs) (EliA CCP; Phadia AB, Uppsala, Sweden); rheumatoid factor (RF) IgM (N Latex RF; Siemens AG, Munich, Germany); hematological inflammatory indices (neutrophils/lymphocytes (N/L), monocytes/lymphocytes (M/L), platelets/lymphocytes (P/L)) [25]; circulating calprotectin (Eurospital, Trieste, Italy); soluble urokinase plasminogen activator receptor (suPAR) (CHORUS suPAR; Diesse Diagnostica Senese SpA, Monteriggioni, Italy); determination of functional classical, MBL, and alternative complement pathways (WIESLAB^®^ Complement system Screen, Euro Diagnostica AB, Malmö, Sweden); circulating cytokines TNFα (Human TNF-alpha Quantikine Immunoassay; R&D Systems Inc., Minneapolis, MN, USA), IL-6 (Human IL-6 Instant Enzyme-linked Immunosorbent assay; eBioscience, Bender MedSystem GmbH, Vienna, Austria), CD3^+^, CD3^+^CD4^+^, CD3^+^CD8^+^, CD3^+^CD4^+^/CD3^+^CD8^+^, CD19^+^, and NKCD3^−^/CD56^+^CD16^+^; and lymphocyte percentages and absolute counts in peripheral whole blood (BD FACS Canto II flow cytometer; Biosciences, San Jose, CA, USA). As regards safety, lipid parameters, total cholesterol (TC), LDL-cholesterol, HDL-cholesterol, triglycerides (TGs), atherogenic index, liver enzymes AST and ALT, creatinine value, and adverse events were evaluated at baseline and after 12 months. The study, involving human participants, was reviewed and approved by the GISEA Project Ethics Review Board on 22 September 2020 (Code of Ethics 6496_OSS). Written informed consent was not required for participation in this study in accordance with national legislation and institutional requirements.

### Statistical Analysis

Since the data have a Gaussian distribution, we applied descriptive statistics, utilizing average and standard deviation (SD) for each item, at baseline and at each time point. The *t*-test was used to check statistical differences in the data between baseline and different time points.

A *p*-value of less than 0.05 was considered statistically significant. Statistical analysis was performed by © 2023 MedCalc Software Ltd. (v22.021, Acacialaan 22, 8400 Ostend, Belgium).

## 3. Results

Table 2 shows the clinimetric parameters at baseline and after 3, 6, and 12 months of treatment with Baricitinib, Filgotinib, Tofacitinib, and Upadacitinib. All treatments demonstrated statistically significant decreased DAS28 and CDAI scores [23,24], as well as tender and swollen joint counts and visual analog scale (VAS), health assessment questionnaire (HAQ), and patient global assessment (PGA) scores after 3, 6, and 12 months of treatment. Differences in laboratory parameters are reported in Table 3. All treatments exhibited comparable responses, with statistically significant decreases observed in circulating levels of calprotectin, TNFα, and IL-6 for all drugs after 12 months of treatment. In addition, suPAR values exhibited significant differences at baseline and after 12 months of treatment for Filgotinib: 4.87 ± 4.53 vs. 3.61 ± 0.9 (0.009) and Upadacitinib: 6.64 ± 7.12 vs. 4.06 ± 3.61 (0.0003), while no statistically significant differences were found for Baricitinib: 3.4 ± 0.1 vs. 3.78 ± 0.1 and Tofacitinib: 3.95 ± 1.77 vs. 2.58 ± 0.1. Liver enzyme, lipid profile, creatinine, and hemoglobin values at baseline and after 12 months of treatment are reported in Table 4. The TC/HDL-C ratio (atherogenic index) showed significant differences in the comparison: Baricitinib vs. Filgotinib (0.0012), Filgotinib vs. Tofacitinib (0.0095), and Filgotinib vs. Upadacitinib (0.0001); furthermore, the LDL-C/HDL-C ratio in the Filgotinib group was not modified (2.37 ± 0.45 vs. 2.35 ± 2.13 (NS)) after 12 months of treatment. Finally, we report the adverse events after 12 months of treatment with Baricitinib, Filgotinib, Tofacitinib, and Upadacitinib in Table 5. Thrombotic events (VTEs) and major adverse cardiovascular events (MACEs) accounted for 1% of adverse events after Baricitinib treatment. *Herpes zoster* reactivation accounted for 1% of adverse events after Filgotinib and Tofacitinib treatment, while non-melanoma skin cancer (NMSC) accounted for 1% of adverse events after Upadacitinib treatment.

## 4. Discussion

Based on the current scientific evidence, the ELECTRA-*i* study is the first real-life monocentric study that has compared the efficacy and safety of the four JAKis currently approved in Italy for the treatment of RA for at least one year. In a prior prospective study, 446 patients diagnosed with RA were enrolled and treated with Baricitinib across 11 Italian centers. The patients were evaluated at baseline and after 3, 6, and 12 months of treatment and were classified into bDMARD-naïve and bDMARD-insufficient responders (IRs). A subanalysis differentiated the effects of MTX and oral glucocorticoid use (OGC). The cohort included 150 (34%) bDMARD-naïve patients and 296 (66%) bDMARD-IR patients, including 217 (49%) using Baricitinib monotherapy. Considering DAS28-CRP as the primary outcome, at 3 and 6 months, 114/314 (36%) and 149/289 (51.6%) patients achieved remission, while 62/314 (20%) and 46/289 (15.9%) had low disease activity (LDA), respectively; furthermore, at 12 months, 81/126 (64%) were in remission and 21/126 (17%) had LDA [26]. A second study was conducted to evaluate the retention rate in 23 Italian tertiary rheumatology centers. The study had a treatment duration of up to 48 months for all patients included in the analysis. The analysis of data from 213 patients revealed that the retention rate of Tofacitinib was 86.5% (95% CI: 81.8–91.5%) after 12 months, 78.8% (95% CI: 78.8–85.2%) after 24 months, 63.8% (95% CI: 55.1–73.8%) after 36 months, and 59.9% (95% CI: 55.1–73.8%) after 48 months from the start of treatment. Among the analyzed factors, no predictive indicators were identified for the retention rate of Tofacitinib [27]. A third recent retrospective study evaluated patients with RA who received a JAKi (Tofacitinib, Baricitinib, Upadacitinib, or Filgotinib) from four tertiary care centers in Milan (Italy). Six hundred and eighty-five patients were included and received Baricitinib (48%), Tofacitinib (31%), Upadacitinib (14%), or Filgotinib (7%), which in 47% were innovative first-line treatments before a biologic. Among a total of 1137 patients, there was one reported stroke and 123 adverse events of special interest (AESIs), accounting for 18% of the total, which included three deaths attributed to serious infections. A higher frequency of adverse events of special interest (23%) was observed among patients with a higher cardiovascular risk [28].

The data emerging from the ELECTRA-*i* study highlighted, as regards clinimetry, that there are no differences in efficacy among the four JAKis. A recent study conducted an adjusted indirect comparison (IC) of randomized clinical trials using Bucher’s method along with an IC and a mixed calculator. The endpoints were C-reactive protein levels and DAS28-CRP and American College of Rheumatology-20 (ACR20) scores. Equivalence was assessed using the Equivalent Therapeutic Alternatives (ETA) guidelines. Of 133 potentially relevant studies, 4 were included. The CI showed no statistically significant differences among the four JAKs regarding DAS28-CRP < 3.2. The results were similar in terms of ACR20, except for Tofacitinib which showed lower efficacy compared to Upadacitinib (RAR: −18.4% [95% CI: −33.4 to −3.5], *p* = 0.0157). Clinically relevant differences were found for Tofacitinib vs. Upadacitinib in both endpoints and for Baricitinib vs. Upadacitinib in both endpoints of DAS28-CRP [29]. A retrospective study recruited 179 patients with RA treated with Baricitinib (2–4 mg/day) or Tofacitinib (10 mg/day). A total of 74 patients received treatment with Baricitinib, while 105 were treated with Tofacitinib. Among them, 83.24% were women, with a median (IQR) age of 56.0 (53.0–56.0) years and a disease duration of 12.0 (6.0–12.0) months. No differences in the rate of LDA were found between the Baricitinib and Tofacitinib treatment groups. The only difference observed was a significantly lower VAS in the Baricitinib group (*p* < 0.05) [30]. Moreover, a Bayesian network meta-analysis included information from direct and indirect comparisons of randomized controlled trials examining remission (DAS28-CRP < 2.6) and LDA (DAS28-CRP ≤ 3.2) after treatment with Tofacitinib, Baricitinib, Upadacitinib, Filgotinib monotherapy, and MTX in DMARD-naïve patients with RA. Four randomized controlled trials, involving 2185 patients, demonstrated that treatment with Tofacitinib, Baricitinib, Upadacitinib, and Filgotinib resulted in a significantly higher remission rate compared to treatment with MTX (odds ratio [OR] = 4.13, 95% CI = 2.88–6.02; OR = 2.12, 95% CI = 1.17–4.13; OR = 1.95, 95% CI = 1.10–3.50; OR = 1.79, 95% CI = 1.27–3.53). The classification probability, based on the evaluation of the surface under the cumulative classification curve, indicated that Upadacitinib 15 mg had the highest probability of achieving remission (SUCRA = 0.985), followed by Tofacitinib 5 mg (SUCRA = 0.574), Baricitinib 4 mg (SUCRA = 0.506), Filgotinib 200 mg (SUCRA = 0.431), and MTX (SUCRA = 0.004) [31]. When assessing effectiveness based on laboratory parameters, our study revealed notable discrepancies in the reduction in suPAR levels following treatment with Filgotinib and Upadacitinib. Both JAKis exhibited a decrease in circulating levels of suPAR [32]. In recent years, urokinase-type plasminogen activator (uPA) protease has been strongly implicated in the pathogenetic process and in the progression of cartilage damage in RA. This physiological process regulates several cellular pathways, including cytokine secretion, cell activation/migration, and fibrinolysis [33,34]. All of these processes begin with an interaction between uPA and its receptor uPAR, which causes tissue remodeling and T cell activation [35]. Moreover, increased uPA expression and decreased tissue plasminogen activator (tPA) expression have been related to the severity of RA disease [36]. Moreover, the activity of synovial cells, including macrophages, fibroblast-like synoviocytes (FLSs), chondrocytes, and endothelial cells, is modulated by the interaction between uPA and uPAR. This interaction enables these cells to secrete various cytokines, chemokines, and growth factors that influence the prognosis of RA [37]. In the absence of macrophage colony-stimulating factor (M-CSF), uPA/uPAR expression suppresses osteoclast differentiation/formation via upregulation of adenosine monophosphate-activated protein kinase (AMPK) [38]. Conversely, other data have demonstrated that in the presence of M-CSF, uPAR promotes osteoclast differentiation via a PI3K/Akt-dependent mechanism [39]. Moreover, other transcription factors (TFs) that it can activate include nuclear factor kappa B (NFKB) and nuclear factor activator of T cells 1 (Nfatc1) [40]. From this perspective, considering the growing utilization of suPAR as a biomarker for monitoring Systemic Chronic Inflammation (SCI) [41], we investigated the effects due to uPA/uPAR interaction in the immune cells involved in RA onset and progression [42]. Furthermore, it has been observed that serum levels of suPAR correlate with disease activity in early RA and reflect joint damage over time [43]. It is possible that the different selectivity on JAK-1 demonstrated for Filgotinib and Upadacitinib could influence this different behavior compared to Tofacitinib and Baricitinib on suPAR [3]. In terms of safety, the data revealing variances between Filgotinib and Baricitinib, as well as between Upadacitinib and Tofacitinib, regarding the atherogenic indices TC/HDL-C and LDL-C/HDL-C are intriguing and could potentially distinguish Filgotinib from other JAKis. This category of drugs not only inhibits cell signaling via the JAK/STAT pathway but also exerts cellular metabolic effects, such as reducing mitochondrial membrane potential, mitochondrial mass, and reactive oxygen species (ROS) levels and inhibiting metabolic genes in synovial tissue [44], as well as modifying systemic lipid metabolism. HDL-C and LDL-C are significantly increased after treatment with Tofacitinib and Baricitinib compared with baseline values and other DMARDs, as shown in RA randomized controlled trials [45,46,47,48], an effect that can be reverted by statins [45]. JAKis also improve HDL function by increasing the activity of lecithin–cholesterol acyltransferase (LCAT; an enzyme that converts free cholesterol to cholesterol esters and supports cholesterol efflux to lipoproteins), increasing HDL efflux capacity [45,46]. Other effects such as alterations in lipoprotein size and content have been described [48,49]. Although treatment with Upadacitinib increases both LDL-C and HDL-C levels, it had no significant effects on cardiovascular risk during a 52-week follow-up [50]. A recent systematic review and network meta-analysis has been performed using randomized controlled trials in RA sourced from PubMed, Medline, Embase, and the Cochrane Controlled Trials Register. The primary outcome was the mean change in HDL-C and LDL-C levels from baseline. The mean treatment differences and range of effects of various JAKis on HDL-C and LDL-C levels were estimated. Based on the data from 18 unique studies involving five approved JAKis and 6697 patients with RA (JAKi = 3341, placebo = 3356), the use of these inhibitors led to a mean increase of 8.11 mg/dL in HDL levels from baseline and a mean increase of 11.37 mg/dl in LDL levels from baseline. The risk of cardiovascular disease did not differ significantly between patients who received JAKis and those who received a placebo or active agents [51]. The better selectivity of Filgotinib on JAK-1 in the absence of activity on JAK-2 could determine an absence of action on leptin, maintaining a stable satiety. Moreover, the action on the lipid profile is only mediated by an IL6-mediated inflammatory mechanism with Tocilizumab [52]. Previously, an absence of alterations in lipid composition was also noted in a multicenter observational study involving 120 patients from rheumatology centers in Tuscany and Umbria (Italy) [53]. A recent review of the literature shows that differences between the various JAKis can be highlighted in terms of selectivity and adverse events [54].

## 5. Conclusions

The findings from the ELECTRA-*i* study corroborate the conclusions drawn from meta-analysis results regarding the comparable efficacy of JAKis. On the other hand, the real-life data show differences between the various JAKis concerning lipid metabolism and the atherogenic index. These differences may be elucidated by variations in JAK selectivity among the different inhibitors.

The potential limitations of our study include the small sample size and the monocentric nature of the study, as well as the unequal distribution of patients across the four JAKis. However, despite these limitations, our study unequivocally demonstrates differences in terms of selectivity, which could have a substantial impact on efficacy and lipid metabolism outcomes.

## Figures and Tables

**Table 1:** Baseline characteristics of the population.

	Baricitinib	Filgotinib	Tofacitinib	Upadacitinib
Previous MACE	5.80%	0	0	6.60%
Diabetes	0	3.12%	0	8.88%
Hypertension	29.40%	46.87%	28.57%	42.22%
Disease duration	107.41 ± 65.96	101.75 ± 95.76	74.23 ± 56.39	88.04 ± 102.25
Body weight	73.52 ± 13.96	68.31 ± 15.82	70.14 ± 11.95	67.62 ± 12.13
BMI	26.43 ± 3.72	25.49 ± 5.57	25.02 ± 3.41	24.82 ± 4.29
MTX	41.17%	40.62%	0.38%	26.66%
Statin	17.64%	3.12%	0	25.90%
Steroid dose	0.58 ± 1.66	2.46 ± 2.34	1.19 ± 2.18	1.28 ± 2.12
Sex	F 82.4%/M 17.6%	F 96.88%/M 3.12	F 90.48/M 9.52	F 93.4/M 6.6
Age	61.4 ± 14.25	67.21 ± 13.11	57.38 ± 16.1	63.26 ± 13.55
Smoke	5.80%	9.37%	4.76%	0.11%
Hormone therapy	0	0	4.47%	2.20%
ACPA	100%	100%	100%	100%
RF	100%	100%	100%	100%
First-line therapy	17.64%	18.75%	47.61%	20%
1 bDMARD	70.6%	25%	38.1%	13.35%
2 bDMARDs	5.88%	31.25%	4.76%	48.88%
3 bDMARDs	5.88%	21.88%	9.53%	11.11%
4 bDMARDs	0%	3.12%	0%	6.66%

**Table 2:** Clinimetric parameters for each treatment group at baseline and 3, 6, and 12 months.

TEN JOINTS	Bas	3 m	6 m	12 m	Bas vs. 3 m	Bas vs. 6 m	Bas vs. 12 m	3 m vs. 6 m	3 m vs. 12 m	6 m vs. 12 m
B	5.82 ± 1.5	1.81 ± 0.54	1 ± 0.1	0.92 ± 0.61	0.0001	0.0001	0.0001	0.0001	0.0001	0.0001
F	5.59 ± 1.31	2.06 ± 0.69	1.16 ± 0.53	0.96 ± 0.79	0.0001	0.0184	0.0001	0.0001	0.0001	0.0001
T	5.71 ± 1.18	1.61 ± 0.76	1 ± 0	1.05 ± 0.8	0.0001	0.0001	0.0001	0.0001	0.0001	0.0001
U	5.62 ± 1.43	1.52 ± 0.64	1.82 ± 1.52	1.39 ± 1.11	0.0001	0.0001	0.0001	0.0001	0.0001	0.0001
**SW JOINTS**										
B	3.82 ± 0.72	1.68 ± 0.47	1 ± 0.1	0.92 ± 0.61	0.0032	0.0001	0.0001	0.0001	0.0001	0.0001
F	4.03 ± 0.57	1.6 ± 0.67	1.16 ± 0.53	0.96 ± 0.79	0.0001	0.0001	0.0001	0.0001	0.0001	0.0001
T	3.8 ± 0.51	1.42 ± 0.6	1 ± 0	1.05 ± 0.8	0.0002	0.0001	0.0001	0.0001	0.0001	0.0001
U	3.88 ± 0.8	1.1 ± 0.7	1.77 ± 1.39	1.36 ± 1.11	0.0001	0.0001	0.0001	0.0001	0.0001	0.0001
**VAS**										
B	32.35 ± 9.03	10 ± 3.16	9.06 ± 2.01	5 ± 0.1	0.0001	0.0001	0.0001	0.0001	0.0001	0.0001
F	31.87 ± 8.59	12 ± 5.5	11.33 ± 5.07	5 ± 0	0.0001	0.0001	0.0001	0.0001	0.0001	0.0001
T	31.9 ± 8.72	13.68 ± 6.83	7.5 ± 2.57	5.83 ± 3.53	0.0001	0.0001	0.0001	0.0001	0.0001	0.0001
U	32.11 ± 7.72	16.5 ± 6.62	8.57 ± 6	7.42 ± 0.21	0.0001	0.0001	0.0001	0.0001	0.0001	0.0001
**HAQ**										
B	1.05 ± 0.31	0.73 ± 0.17	0.5 ± 0	0.5 ± 0	0.0001	0.0001	0.0001	0.0001	0.0001	NS
F	1.03 ± 0.27	0.67 ± 0.18	0.53 ± 0.1	0.53 ± 0.13	0.0001	0.0001	0.0001	0.0001	0.0001	NS
T	1 ± 0.2	0.63 ± 0.17	0.55 ± 0.1	0.56 ± 0.16	0.0001	0.0001	0.0001	0.0001	0.0001	0.0001
U	1.11 ± 0.26	0.61 ± 0.13	0.61 ± 0.25	0.64 ± 0.21	0.0001	0.0001	0.0001	NS	0.0001	0.0001
**PGA**										
B	32.35 ± 9.03	11.25 ± 4.65	9.37 ± 1.7	5 ± 0	0.001	0.0001	0.0001	0.0001	0.0001	0.001
F	29.84 ± 8.08	13.66 ± 5.56	11.33 ± 5.07	5 ± 0	0.001	0.0001	0.0001	0.0001	0.0001	0.001
T	30.47 ± 5.89	12.63 ± 4.52	7.5 ± 2.57	5.83 ± 3.53	0.001	0.0001	0.0001	0.0001	0.0001	0.001
U	32.77 ± 7.65	13.75 ± 4.9	8.57 ± 4.46	7.42 ± 8.2	0.001	0.0001	0.0001	0.0001	0.0001	0.001
**DAS28**										
B	4.44 ± 0.64	2.81 ± 0.25	2.06 ± 1.7	1.89 ± 0.4	0.0001	0.0001	0.0001	0.0001	0.0001	0.0001
F	4.44 ± 0.44	2.72 ± 0.52	2.11 ± 0.71	1.82±0.42	0.0001	0.0001	0.0001	0.0001	0.0001	0.0001
T	4.46 ± 0.43	2.52 ± 0.83	1.96 ± 0.55	1.97 ± 0.59	0.0001	0.0001	0.0001	0.0001	0.0001	0.0001
U	4.39 ± 0.49	2.8 ± 0.46	2.02 ± 0.48	2.27 ± 0.83	0.0001	0.0001	0.0001	0.0001	0.0001	0.0001
**CDAI**										
B	19.17 ± 3.82	13.18 ± 1.79	10 ± 1.77	9.07 ± 1.32	0.0001	0.0001	0.0001	0.0001	0.0001	0.0001
F	19.12 ± 2.75	12.93 ± 2.3	9.73 ± 2.54	9.21 ± 0	0.0001	0.0001	0.0001	0.0001	0.0001	0.0001
T	19.61 ± 3.47	12.42 ± 5.19	10 ± 2.82	9.77 ± 2.71	0.0001	0.0001	0.0001	0.0001	0.0001	0.0001
U	19.04 ± 3.48	9.62 ± 3.35	9.48 ± 2.35	10.6 ± 3.2	0.0001	0.0001	0.0001	0.0001	0.0001	0.0001

Bas = baseline; m = months; B = Baricitinib; F = Filgotinib; T = Tofacitinib; U = Upadacitinib; NS = not significant. Ten Joints = tender joints; Sw Joints = swollen joints.

**Table 3:** Blood markers in evolution in four populations treated with Baricitinib, Filgotinib, Tofacitinib, and Upadacitinib. The comparison is baseline versus 12 months.

N/L	Bas	12 m	Bas vs. 12 m	ESR	Bas	12 m	Bas vs. 12 m
B	2.73 ± 1.09	2.77 ± 1.07	0.0001	B	27.35 ± 21.73	43.64 ± 27.75	0.017
F	2.8 ± 1.37	2.49 ± 0.85	0.0001	F	40.34 ± 28.89	37.06 ± 21.61	0.0001
T	2.16 ± 0.9	2.52 ± 1.22	0.0001	T	33.04 ± 21.2	28.55 ± 15.25	0.0001
U	2.25 ± 1.27	2.83 ± 1.26	0.0001	U	35.86 ± 24.63	31.51 ± 20.07	0.0001
**Plt/L**	**Bas**	**12 m**	**Bas vs. 12 m**	**CRP**	**Bas**	**12 m**	**Bas vs. 12 m**
B	208.05 ± 107.47	206.85 ± 60.97	0.0001	B	0.31 ± 0.36	0.4 ± 0.35	0.0001
F	188.15 ± 79.13	189.31 ± 56.77	0.0001	F	1.12 ± 1.39	0.32 ± 0.32	0.0001
T	158.47 ± 78.42	163.8 ± 37.55	0.0001	T	0.6 ± 0.51	0.32 ± 0.23	0.0001
U	169 ± 83.8	181.18 ± 76.35	0.0001	U	1.12 ± 1.88	0.6 ± 0.54	0.0001
**M/L**	**Bas**	**12 m**	**Bas vs. 12 m**	**IL-6**	**Bas**	**12 m**	**Bas vs. 12 m**
B	0.33 ± 0.16	0.36 ± 0.14	0.0001	B	4.77 ± 4.11	3.19 ± 0.72	0.0001
F	0.36 ± 0.16	0.34 ± 0.14	0.0001	F	11.8 ± 18.66	3.21 ± 1.15	0.0001
T	0.26 ± 0.11	0.26 ± 0.13	NS	T	3.33 ± 1.12	3 ± 0.1	0.0001
U	0.27 ± 0.14	0.32 ± 0.14	0.0001	U	11.2 ± 22.26	4.92 ± 4.72	0.0004
**CD8**	**Bas**	**12 m**	**Bas vs. 12 m**	**TNFα**	**Bas**	**12 m**	**Bas vs. 12 m**
B	484.11 ± 273.8	378.14 ± 233.41	0.0001	B	34.52 ± 7.9	15.6 ± 3.69	0.0001
F	367.9 ± 152.86	401.27 ± 139.89	0.0001	F	21.74 ± 23.84	15.6 ± 7.23	0.0001
T	462.28 ± 224.46	380.16 ± 183.81	0.0001	T	41.61 ± 56.92	15.58 ± 0.7	0.0001
U	460 ± 296.25	352.15 ± 234.62	0.0001	U	22.95 ± 24.45	16.2 ± 3.2	0.0001
**CD3**	**Bas**	**12 m**	**Bas vs. 12 m**	**MRP**	**Bas**	**12 m**	**Bas vs. 12 m**
B	1298.23 ± 526.9	1151.21 ± 548.67	0.0001	B	2.33 ± 0.89	2.11 ± 0.5	0.0001
F	1224.06 ± 412.99	1150.93 ± 326.44	0.0001	F	5.72 ± 17.01	2.1 ± 0.57	0.0001
T	1500 ± 404.76	1212.22 ± 308.92	0.0001	T	1.97 ± 0.49	1.77 ± 0.31	0.0001
U	1526.25 ± 821	1419.69 ± 797.76	0.0001	U	3.37 ± 5.19	2.24 ± 1.22	0.0001
**CD56**	**Bas**	**12 m**	**Bas vs. 12 m**	**suPAR**	**Bas**	**12 m**	**Bas vs. 12 m**
B	265.23 ± 160.28	309.07 ± 234.26	0.0001	B	3.4 ± 0.1	3.78 ± 0.1	NS
F	241.4 ± 126.57	266.24 ± 232.01	0.0001	F	4.87 ± 4.53	3.61 ± 0.9	0.009
T	305.71 ± 143.03	149.55 ± 103.12	0.0001	T	3.95 ± 1.77	2.58 ± 0.1	NS
U	285.92 ± 221.61	220.09 ± 165.75	0.0001	U	6.64 ± 7.12	4.06 ± 3.61	0.0003
**CD4**	**Bas**	**12 m**	**Bas vs. 12 m**	**MBL**	**Bas**	**12 m**	**Bas vs. 12 m**
B	811.82 ± 392.3	744.71 ± 309.29	0.0001	B	51.05 ± 31.21	71.5 ± 23.59	0.0001
F	850.65 ± 318.52	709.79 ± 206.64	0.0001	F	61.68 ± 42.97	63.83 ± 23.19	0.0001
T	1011.57 ± 273.57	786.05 ± 136.51	0.0001	T	49.92 ± 36.92	56.35 ± 14.58	0.0019
U	1050.93 ± 641.81	971 ± 684.94	0.0001	U	50.33 ± 47.94	70.97 ± 59.04	0.0001
**CD19**	**Bas**	**12 m**	**Bas vs. 12 m**	**CL**	**Bas**	**12 m**	**Bas vs. 12 m**
B	218.64 ± 143.38	174.92 ± 179.4	0.0001	B	120.76 ± 10.68	123.57 ± 31.59	0.0001
F	143.31 ± 110.28	130.2 ± 71.43	0.0001	F	121.54 ± 27.94	123.58 ± 26.56	0.0001
T	268.38 ± 145.32	170.88 ± 61.52	0.0001	T	116.66 ± 16.23	132.88 ± 20.53	0.0001
U	182.68 ± 131.87	170.21 ± 106.74	0.0001	U	110.75 ± 20.38	129.75 ± 23.97	0.0001
				**Alt**	**Bas**	**12 m**	**Bas vs. 12 m**
				B	104.82 ± 19.98	102.57 ± 13.27	0.0001
				F	94.09 ± 25.93	95 ± 17.4	NS
				T	92.14 ± 11.12	100.76 ± 9.6	0.0001
				U	99.59 ± 23.2	96.12 ± 16.26	0.0001

Bas = baseline; m = months; B = Baricitinib; F = Filgotinib; T = Tofacitinib; U = Upadacitinib, NS = not significant.

**Table 4:** Liver enzyme, lipid profile, creatinine, and hemoglobin values at baseline and after 12 months in each group of treatment.

AST	Baseline	12 Months	Bas. Vs. 12 Months (*p*-Value)
Baricitinib	24.11 ± 8.1	20.28 ± 4	NS
Filgotinib	22.71 ± 8.55	22.96 ± 5.86	NS
Tofacitinib	24.23 ± 9.97	21.44 ± 3.74	NS
Upadacitinib	21.42 ± 5.49	22.63 ± 7.78	NS
**Cholesterol**			
Baricitinib	226.41 ± 23.49	245 ± 17.59	0.0001
Filgotinib	212.03 ± 32.05	210.03 ± 23.64	0.0001
Tofacitinib	222 ± 49.12	217.94 ± 33.92	0.0001
Upadacitinib	208.88 ± 24.87	236.87 ± 25.35	0.0001
**LDL/HDL ratio**			
Baricitinib	2.44 ± 0.53	2.57 ± 0.59	0.0001
Filgotinib	2.37 ± 0.45	2.35 ± 2.13	NS
Tofacitinib	2.27 ± 0.36	2.55 ± 2.64	0.0001
Upadacitinib	2.35 ± 0.37	2.64 ± 0.56	0.0001
**ALT**			
Baricitinib	22.11 ± 6.2	24.5 ± 7.7	0.0001
Filgotinib	20.5 ± 8.18	19.39 ± 4.66	0.0001
Tofacitinib	24.66 ± 14.99	19.38 ± 6.74	0.0001
Upadacitinib	21.02 ± 8.01	22.33 ± 9.92	0.0001
**LDL**			
Baricitinib	134.64 ± 17.59	159.5 ± 21.79	0.001
Filgotinib	134 ± 15.86	128 ± 27.25	0.001
Tofacitinib	139.66 ± 33.98	132.27 ± 33.92	0.001
Upadacitinib	130.33 ± 14.81	151.51 ± 27.98	0.001
**Creatinine**			
Baricitinib	0.82 ± 0.13	0.81 ± 0.14	0.0001
Filgotinib	0.66 ± 0.2	0.7 ± 0.1	0.0001
Tofacitinib	0.78 ± 0.22	0.73 ± 0.14	0.0001
Upadacitinib	0.77 ± 0.22	0.76 ± 0.15	0.0001
**HDL**			
Baricitinib	57 ± 10.74	63.57 ± 21.79	0.0001
Filgotinib	55.18 ± 15.86	60.35 ± 24.86	0.0001
Tofacitinib	61.47 ± 10.02	51.5 ± 12.82	0.0001
Upadacitinib	55.97 ± 7.12	58.69 ± 10.57	0.0001
**Hb**			
Baricitinib	13.44 ± 1.51	12.79 ± 1.83	0.046
Filgotinib	12.9 ± 1.51	12.9 ± 1.77	0.0001
Tofacitinib	13.14 ± 1.62	13.41 ± 1.6	0.0001
Upadacitinib	13.05 ± 1.25	12.73 ± 1.23	0.0001
**Total Cholesterol/HDL**			
Baricitinib	4.11 ± 0.9	3.94 ± 0.74	NS
Filgotinib	3.9 ± 0.65	3.24 ± 0.84	NS
Tofacitinib	3.53 ± 0.59	5.23 ± 4.9	NS
Upadacitinib	3.79 ± 0.68	4.13 ± 0.66	NS

NS—not significant.

**Table 5:** Adverse events after 12 months.

Adverse Events—Number (%)	Baricitinib	Filgotinib	Tofacitinib	Upadacitinib
**MACE**	1 (5.88)	0	0	0
**VTE**	1 (5.88)	0	0	0
**HZ**	0	1 (3.12)	1 (4.76)	0
**NMSC**	0	0	0	1 (2.22)
**Cancer**	0	0	0	0

HZ—Herpes zoster, VTE—venous thromboembolism, MACE—major adverse cardiovascular event, NMSC—no-melanoma skin cancer.

## Data Availability

The raw data supporting the conclusions of this article will be available from the authors without undue reservation.

## Abbreviations

Rheumatoid arthritis	RA
Janus kinase	JAK
Signal transducers and activators of transcription	STAT
Biological disease-modifying antirheumatic drugs	bDMARDs
Conventional synthetic disease-modifying antirheumatic drugs	csDMARDs
JAK inhibitors	JAKis
Number of tender joints	NTJs
Number of swollen joints	NSJs
Visual analog scale	VAS
Global assessment	GA
Health assessment questionnaire	HAQ
patient global assessment	PGA
Disease Activity Score	DAS28
Soluble urokinase plasminogen activator receptor	suPAR
Total cholesterol	TC
LDL-cholesterol	LDL-C
HDL-cholesterol	HDL-C
Triglycerides	TGs
